# BMC health services research title: the 2020 blast in the port of Beirut: can the Lebanese health system “build back better”?

**DOI:** 10.1186/s12913-020-05906-y

**Published:** 2020-11-12

**Authors:** Michel D. Landry, Mohamad Alameddine, Tiago S. Jesus, Saydeh Sassine, Elie Koueik, Sudha R. Raman

**Affiliations:** 1 Duke University, Durham, NC USA; 2 Mohammed Bin Rashid University of Medicine and Health Sciences, Dubai, UAE; 3 American University of Beirut, Beirut, Lebanon; 4 Institute of Hygiene and Tropical Medicine - NOVA University of Lisbon, Lisbon, Portugal; 5 Lebanese German University, Jounieh, Lebanon; 6 Order of Physiotherapists in Lebanon, Beirut, Lebanon

## Abstract

The August 2020 explosion in Lebanon resulted in casualties, injuries, and a great number of internally displaced persons. The blast occurred during an economically and politically complex time in the country. Given multiple and competing post-explosion reconstruction priorities, in ths editorial we briefly examine the requirements for a build back better scenario.

## Main text

**Fig. 1:**
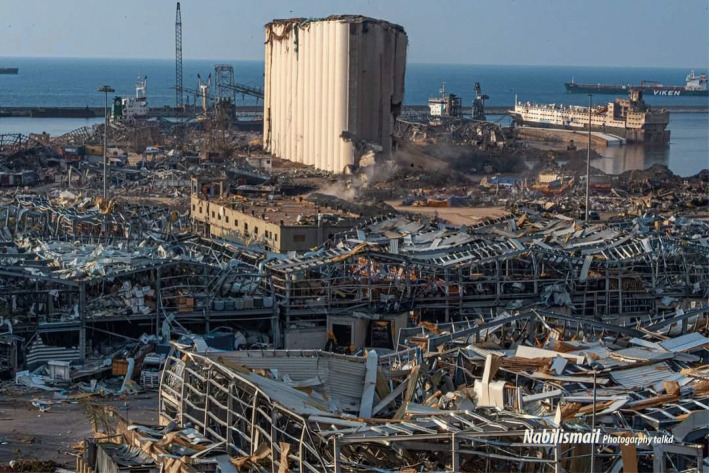
The Port of Beirut (August 5, 2020). (Courtesy of Nabil Ismail)

On August 4, 2020, a cache of approximately 2750 metric tons of ammonium nitrate precariously stored in the port of Beirut, Lebanon ignited and set off a massive high order blast that destroyed large parts of the ancient city. The strength of the blast was is considered one of the largest explosions recorded in modern history [1, 2]. While the motives behind storing a sizeable accumulation of highly explosive materials for years in a densely populated urban area will be a matter for future investigations, in this editorial, we elaborate on the health outcomes and health systems effects of the blast that resulted in over 200 casualties, 7000 injured and over 300,000 internally displaced persons [3] (Fig. 1).

### The Lebanese context

Lebanon, a small upper-middle-income Mediterranean nation, is geopolitically complex. First, a 15-year civil war from 1975 to 1990 resulted in a precarious and complex balance of shared political leadership along with religious affiliations which have reportedly lead to significant corruption (Lebanon is ranked 137 out of 180) and noteworthy mismanagement, placing into question culpability for the disaster and even ability to orchestrate recovery efforts [4]. *Second,* the decade-long Syrian conflict created a mass exodus of people seeking refuge, and Lebanon opened its northern border and accepted millions of refugees fleeing violence. The refugees displaced by the Syrian protracted crisis were added to an estimated half a million Palestinian refugees displaced by the Arab-Israeli conflict. As a result, Lebanon currently has the world’s highest ratio of refugees to population, meaning that currently 1 in 4 people who live in Lebanon are refugees. In and of itself, a country that has high numbers of refugees can be a signal of a sovereign nation’s willingness to ‘do its part’; but on the other hand, the influx of such high absolute and proportional numbers has resulted in pronounced political and economic tensions [5]. *Third,* in late 2019, national protests ignited across Lebanon against the government halted the economy, and ultimately triggered the Lebanese currency to devalue by close to 80% in a matter of months, and triggered waves of hyperinflation. Currently an estimated 50% of Lebanese, or about 3.5 million people, live below the poverty line and under conditions of food insecurity and mass unemployment [6]. *Fourth*, while COVID19 has touched almost all parts of the globe, it is also prominent that the pandemic has created disproportional effects in fragile settings such as Lebanon, especially after the devastating blast, where relatively weak public health infrastructure exists. For instance, the International Rescue Committee has reported that the rates of COVID19 infections have increased by 220% in post-explosion Lebanon [7].

Overall, even before the blast, Lebanon had a great number of political, social, and economic crises that have hampered the countries ability to respond to the disaster and plan for the recovery phase. The World Bank Group estimated the cost of recovery to be between USD 3.8 and 4.6 Billion for the physical infrastructure alone, and between USD 2.9 and 3.5 Billion to replenish the erosion in the economic output, and Lebanese officials reported the total cost of the blast to be USD 15 Billion [8]. A global donor initiative led by the French Government pledged $300 Million leaving a significant shortfall in reconstruction aid. A notable factor hampering the flow of international assistance is the hesitation on behalf of the global community, and of many Lebanese themselves, to provide direct emergency financing to the government due to long-standing corruption and mismanagement.

### Anatomy of the blast

**Fig. 2:**
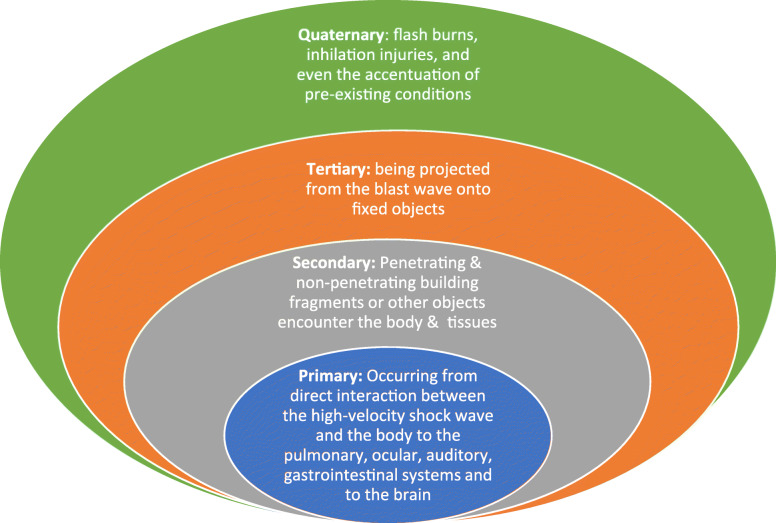
Blast Injury Categories

According to Dahlquist et al. [9], explosions result in “*extensive injuries to multiple locations in the body leading to severe complications such as catastrophic hemorrhage and trauma-induced coagulopathy*”. The emergency response mounted by the Lebanese medical and non-medical (volunteers) communities, amid weak infrastructure and poor resources, was instrumental in saving the lives of many. Blast injuries are generally categorized into four main categories [10, 11], all of which were witnessed after the blast (Fig. 2).

Beyond these categories of injury, the blast has had other effects; for instance, Gourd [12] reported that the physical destruction and broken supply chain in after the blast has meant that, for example, many pediatric cancer patients have lapsed in essential chemotherapies, and that referral to other hospitals is challenged by the increased number of injuries. The data regarding injury type and frequency from the Beirut blast are not readily available as the documentation was generally inadequate. Nevertheless, informal reports compiled by International agencies, suggested that the blast induced injurieswere mainly polytrauma, tendon rupture, upper extremity injuries, ophthalmologic, maxilla-facial and cranial [13]. In the longer term, a bifurcated public and private health system will need to prepare for the increase health and rehabilitation needs among the blast survivors.

Beyond the human toll, the blast damaged key health infrastructure. According to the World Health Organization, following the catastrophic blast, more than half of the health facilities in Beirut were damaged and were ‘non-functional’ thereby limiting the supply of care to meet the spiking health care needs [14]. Additionally, the civil unrest and protests that ignited after the blast also resulted in several injuries and thereby adding to the already unbalanced supply and demand for health services in Beirut. Given the surge in demand, the functional hospitals used a 2 months stock of medical supplies within days of the blast. Moreover, after the explosion, all the resources of the health care sector and that of the community were spontaneously mobilized to help with relief and construction efforts. This may have masked some of the fragmentation and structural weaknesses in the care system, especially with the international flow of relief aids and supplies. Two months after the explosion, the weaknesses surfaced again and the collapse of the health care and social services systems appears to be imminent.

### ‘Build back better’ in the health sector?

The blast highlighted the systemic geopolitical and health system issues that persist in Lebanon. ‘Build Back Better (BBB), a concept that originally evolved from the United Nations’ SENDAI framework and which was adopted by the UN General Assembly in 2015, is an approach that positions disasters as a stimulus to prompt a nation to develop greater ‘resilience.’ While a disaster of this caliber has shed light on the weakness in planning and implementation, it does provide an opportunity to improve the health systems. While the international community can and should play a role, it will be up to Lebanon to decide on the direction. Given the multitude of ‘pre-existing’ financial and infrastructure conditions, we wonder whether the health sector will figure prominently, or whether it will take a back seat in a rebuilding process.

In a 2015 publication on rebuilding communities following disasters, the US Institute of Medicine provides a framework to position public health as a priority [15]. In and of itself, this and other frameworks are a tremendous roadmap, but in the case of Lebanon, it does not necessarily take into account the depth and breadth of the complexities that exist in pre-blast Beirut, all of which have become accentuated. The surge of supply-demand gaps led by the blast (and the COVID-19 pandemic) just shed further light on structural system fragilities that already existed and won’t disappear even after this surge of need has been reduced unless the opportunity is taken to strengthen the fragile system. The road ahead and the BBB strategy in Lebanon’s health sector will be complex due to the global pandemic and other economic and social ‘pre-existing conditions’. This is a critical juncture in the history of the country, a BBB scenario is possible (albeit difficult), yet requires a strategic reengineering of a political system that has institutionalized corruption, favoritism, and sectarianism for decades.

Lebanon is the land of the ancient Phoenecian civilization, which has been continuously inhabited for well over 5000 years. They have survived a great number of sudden-onset and human-constructed disasters over the millenia, and they will survive this tragedy as well. On the one hand, the resilience of the Lebanese is often compared to that of the mythical Phonecian Bird (otherwise known as a Pheoxnix) which does not die, and only emerged from the ashes to survive another day. However, on the other hand, it may be that such robust resilience has cost Lebanon too much aleady, and maybe this event becomes the ‘line in the sand’ towards important reform. If so, can the pre-blast, fragile Lebanese health system be part of that reform, and be built back better? One can be hopeful, albeit unsure.

## Data Availability

Not applicable.
