# Correction to: The 2020 blast in the Port of Beirut: can the Lebanese health system “build back better”?

**DOI:** 10.1186/s12913-020-05966-0

**Published:** 2020-12-03

**Authors:** Michel D. Landry, Mohamad Alameddine, Tiago S. Jesus, Saydeh Sassine, Elie Koueik, Sudha R. Raman

**Affiliations:** 1 Duke University, Durham, NC USA; 2 Mohammed Bin Rashid University of Medicine and Health Sciences, Dubai, UAE; 3 American University of Beirut, Beirut, Lebanon; 4 Institute of Hygiene and Tropical Medicine, NOVA University of Lisbon, Lisbon, Portugal; 5 Lebanese German University, Jounieh, Lebanon; 6 Order of Physiotherapists in Lebanon, Beirut, Lebanon

**Correction to: BMC Health Serv Res 20, 1040 (2020)**

**https://doi.org/10.1186/s12913-020-05906-y**

Following publication of the original article [1], the Editor identified an error in the article title.

The article title currently reads:

**BMC health services research title: the 2020 blast in the port of Beirut: can the Lebanese health system “build back better”?**

The article title should read:

**The 2020 blast in the Port of Beirut: can the Lebanese health system “build back better”?**

The original article has been corrected.
